# A cross-sectional study of *Simulium damnosum* sensu lato breeding sites and species distribution in Sudan savanna, mixed savanna–forest and rainforest regions in Cameroon

**DOI:** 10.1186/s13071-022-05462-w

**Published:** 2022-10-21

**Authors:** Franklin Ayisi, Naniogué Sedou, Stephanie Kouahou Dieunang, Florent Yaya, Edmond François Tchago, Cosmas Ejong Ndellejong, Benjamin Biholong, Daniel Adjei Boakye

**Affiliations:** 1 National Onchocerciasis Control Programme, Ministry of Public Health, Yaoundé, Cameroon; 2 African Regional Postgraduate Programme in Insect Science (ARPPIS), University of Ghana, Legon-Accra, Ghana; 3 WHO/ESPEN Laboratory, Ouagadougou, Burkina Faso; 4 Noguchi Memorial Institute for Medical Research (NMIMR), University of Ghana, Legon-Accra, Ghana; 5 The End Fund, New York, NY USA

**Keywords:** Onchocerciasis, *Simulium damnosum*, Breeding site, Species, Forest, Cameroon

## Abstract

**Background:**

The presence of breeding sites and distribution of species of *Simulium damnosum* sensu lato are critical in understanding the epidemiology of onchocerciasis and evaluating the impact of elimination interventions. Reports on breeding sites and species distribution of members of *S. damnosum* s.l. in Cameroon are scarce and the few ones available date back to more than three decades. The aim of this study is to provide information on *S. damnosum* breeding sites across the rainy (RS) and dry (DS) seasons and the species composition in three different regions in Cameroon: Southwest (SW), Northwest (NW) and North (N).

**Methods:**

A cross-sectional two-season study was carried out in three regions with different ecological characteristics (SW—rainforest; NW—mixed forest–Guinea savanna; N—Sudan savanna). Pre-control onchocerciasis endemicity, relief maps and historical entomological information were used to identify potential rivers for purposive sampling. Sampled larvae were fixed in Carnoy’s solution and sorted, and *S. damnosum* s.l. larvae were stored until identification by cytotaxonomy. Geographical coordinates of potential breeding sites were recorded to produce maps using ArcGIS, while Chi-square tests in SPSS were used to test for any differences between black fly seasonal breeding rates.

**Results:**

A total of 237 potential breeding sites were sampled (RS = 81; DS = 156) and 72 were found positive for *S. damnosum* s.l. The SW had the most positive sites [67 (RS = 24; DS = 43)], with a significant difference in the rate of breeding between the seasons (*P* < 0.05). Among 68 sites visited in both seasons, 16 (23.5%) were positive in one of the two seasons with more sites positive in DS(11) than RS(05), 14 (20.6%) and 38 (55.9%) respectively positive and negative in both seasons. *Simulium damnosum* sensu stricto and *S. sirbanum* were the main species in the N, while *S. squamosum* and *S. mengense* were the predominant species in the NW and SW. *Simulium soubrense* and *S. yahense* were uniquely recorded in the SW.

**Conclusions:**

A comprehensive mapping of breeding sites requires rainy and dry seasons sampling. This study demonstrates that a breeding site survey of *S. damnosum* s.l. is achievable in forest as well as savanna zones. Not all potential breeding sites are actual breeding sites. Observation of *S. soubrense* in the SW indicates changes in species composition over time and could affect onchocerciasis epidemiology in this area.

**Graphical abstract:**

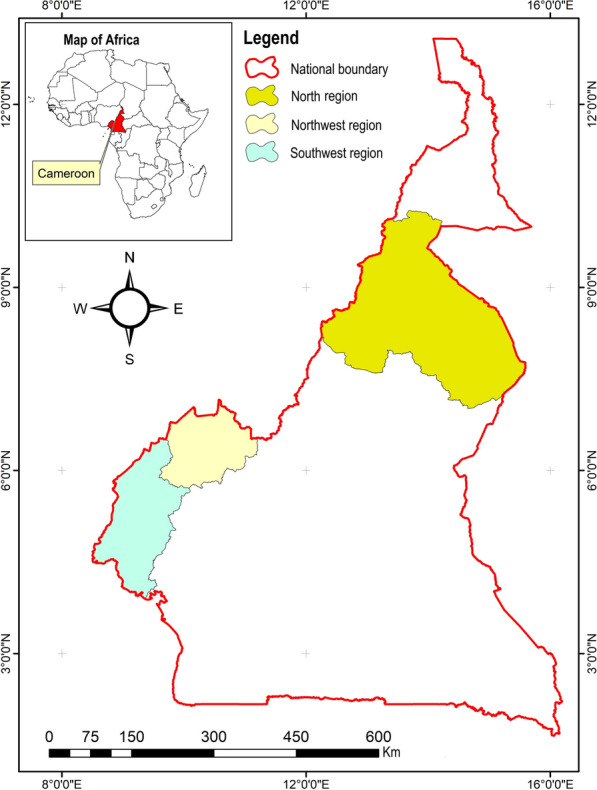

**Supplementary Information:**

The online version contains supplementary material available at 10.1186/s13071-022-05462-w.

## Background

Human onchocerciasis, also called river blindness, is a parasitic infection caused by the filarial nematode *Onchocerca volvulus* and transmitted by female black flies of the genus *Simulium* (Diptera: Simuliidae) [1]. The most important vectors are members of the *Simulium damnosum* complex, which have a wide range throughout Africa and the Middle East [2]. The flies breed in fast-flowing stretches of water, where juvenile stages can be found attached to submerged vegetation, rocks or other substrates.

*Simulium damnosum* sensu lato are the only known vectors of human onchocerciasis in West and Central Africa. The species of this complex vary in their biting cycle and population age structure [3], response to control measures, ecological adaptation and host preference, as well as their ability to transmit the causative agent of human onchocerciasis [4–7]. Members of the *S. damnosum* complex have been categorized into forest and savanna species [8] depending on their predominant ecological preferences.

The transition from control to elimination of onchocerciasis gives rise to the need for reassessment of all previously excluded areas from onchocerciasis interventions during the control era, termed ‘elimination mapping’ [9, 10]. Positive findings from breeding site assessments are important in defining the target population for such mapping [9], given that the presence of vector *Simulium* flies defines the epidemiology of onchocerciasis [11]. In addition, breeding site surveys constitute important first steps in entomological and epidemiological evaluations of interventions in the different stages of onchocerciasis elimination [10].

Among the entomological procedures in the fight against human onchocerciasis, breeding site surveys form the basic foundation, as they are required in most of the phases of onchocerciasis control and elimination. It provides information on where different vector species occur and define communities exposed to different levels of onchocerciasis transmission. However, breeding site surveys are often thought to be impractical or difficult to carry out, especially in forest zones [12]. This has served as a limitation to onchocerciasis control/elimination efforts in forest zones.

The presence and characteristics of breeding sites of *S. damnosum* s.l. may change with seasons and over time either due to pressure from human activity such as deforestation [13] and climate change [14], or due to other anthropogenic or natural causes. This might lead to changes in vector species distribution and consequently the transmission pattern of the disease. Therefore, it is important to regularly update the breeding sites and vector species distribution to detect early changes in the epidemiology of onchocerciasis.

Few studies have documented the breeding sites and species distribution of vectors of onchocerciasis in Cameroon. Available studies date from more than three decades ago [15, 16] and/or were restricted to a particular region, locality or river basin of interest [16–19]. Ten cytoforms of *S. damnosum* s.l. (*S. damnosum* sensu stricto Nile and Volta forms, *S. sirbanum* s.l., *S. mengense*, *S. squamosum* A, B, C, D and E2, and *S. yahense*) have been reported from Cameroon [20], all of which are either confirmed or suspected vectors of human onchocerciasis [21–23].

The geographical distribution of *S. damnosum* s.l. in the country follows the established eco-geography of these vectors, where the savanna cytospecies (*S. damnosum* s.s. and *S. sirbanum*) mostly occupy streams and rivers in the Sudan savanna areas, while the forest cytospecies (*S. squamosum*, *S. yahense* and *S. mengense*) are mostly encountered in the forest and Guinea savanna areas [15, 18, 24]. Nevertheless, *S. mengense* and *S. squamosum* A are widely distributed throughout the country, and often observed in savanna areas sharing the same habitat as the savanna vectors [15, 18]. Likewise, the savanna species, *S. damnosum* s.s., has been observed in the forest Southwest region [17, 25]. Around Mount Cameroon, *S. squamosum* A, C and D occur in sympatry, where hybrids between cytoforms A and C are common [18, 19]. *Simulium squamosum* B has only been found in the Sanaga River [18] while *S. squamosum* E2 has recently been described from the Mbam River [26], a tributary of the Sanaga River.

The present study was therefore carried out to provide an updated map of breeding sites and species distribution of black flies in three administrative regions of Cameroon having different vegetation types—North (Sudan savanna), Northwest (mixed forest–savanna) and Southwest (rainforest). The study also demonstrates the feasibility of carrying out breeding site surveys in forest zones similar to savanna zones.

## Methods

### Study site

**Fig. 1:**
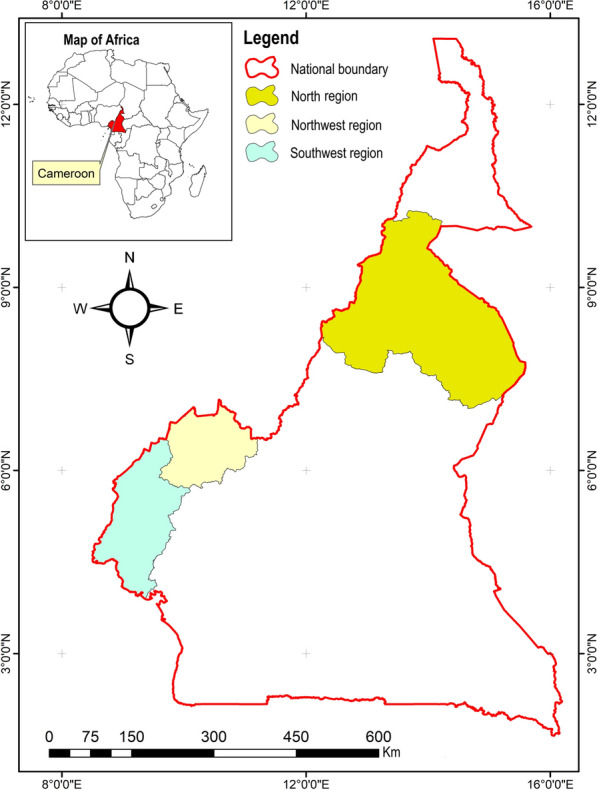
Map of Cameroon showing the three regions in this study

This study took place in three administrative regions of Cameroon, namely, the Southwest (SW), Northwest (NW) and North (N) regions (Fig. 1).

The Southwest region has dense evergreen and montane vegetation and a humid tropical rainforest (that is gradually being degraded for lumbering and agricultural activities). It has an equatorial climate with rainfall between 2000 and 4000 mm per year and a dense seasonal and perennial hydrographic network constituting the Manyu (Munaya), Meme, Mungo and Ndian drainage basins. Part of this region falls within the coastal maritime zone with coastal vegetation consisting of mangroves and tropical rain forest. The topography of the region comprises mountains, hills and valleys that generate fast water currents and natural waterfalls. An abandoned dam (formerly used to generate hydroelectric power in the area) over the Yoke River around Muyuka now serves as a small waterfall with several rapids. Coupled with the long rainy seasons (March–October), this ecology favours the establishment of permanent breeding sites required for continuous onchocerciasis transmission.

The Northwest region falls within the mountain range of Cameroon, characterized by relief of massifs and mountains. The once large forest area has been reduced to mostly grassland and bush savanna, partly because of deforestation for timber, firewood and agriculture, with only a few patches of forest left. The region has a tropical climate characterized by two main seasons: the rainy season, which starts in March and ends in October, and the dry season from November to February. Rainfall varies between 1700 and 3000 mm annually. The main rivers are the Menchum, Bui, Katsina, Kumbi, Momo, Noun and Donga rivers, with several seasonal and perennial tributaries.

The North region falls within the Sudano–Sahelian ecological zone of the country, having a dense hydrographic network of streams/rivers (*mayos*). The streams/rivers are generally seasonal, but for the Vina, Mbere and Benoue rivers. A dam on the Benoue River at Lagdo is used for generating electricity and providing water for irrigation. Aside from the perennial rivers, most of the streams and rivers in the region dry up or are reduced to pools (which later dry up) during the dry season. The region has a short rainy season (July–October) and a long dry season (November–June), with unevenly spatio-temporally distributed mean annual rainfall of about 800–900 mm [27].

### Sample collection

Sampling took place in the months of July and September 2012, and December 2012–February 2013 (taking a break from 22 December 2012–10 January 2013). Potential breeding sites were determined from pre-control REMO (rapid epidemiological mapping of onchocerciasis) infection maps and other historical onchocerciasis information, coupled with a detailed relief map (Additional file 1: Fig. S1) of the study regions. Purposive sampling for *S. damnosum* s.l. larvae was undertaken on the rivers showing potential breeding sites. *S. damnosum* s.l. larvae were collected from trailing vegetation and other submerged substrates (e.g. grass, leaves and rocks) in fast-running water stretches. The larvae were stored in Carnoy’s solution (absolute ethanol and glacial acetic acid mixture of v/v 3:1) and kept cold in an ice chest containing ice blocks until taken to the lab where they were sorted into various *Simulium* species and those of the *S. damnosum* s.l. were stored in the refrigerator for later cytotaxonomic analysis. Larvae of the *S. damnosum* s.l. were identified by the presence of scales (spines) over the dorsal and lateral surfaces of the body and proleg and their characteristic dorsal tubercles [28].

### Cytotaxonomic identification of *S. damnosum* s.l. larvae

The collected larvae were processed for cytotaxonomic identification as described in [29] and identifications made based on the species diagnostic inversions described in [19, 21, 30]. Briefly, larval abdomens were teased open slightly and stained with orcein. Stained salivary glands were extracted under a dissection microscope. Stained salivary gland cells were then gently compressed between a glass slide and cover slip and the banding pattern of the resulting spread of polytene chromosomes examined for inversions.

### Data analysis

SPSS version 25 statistical software was used to perform Chi-square tests to estimate the significance between black fly breeding rate and seasonality.

## Results

### Breeding site surveys

A total of 81 and 156 potential breeding sites were surveyed during the rainy and dry seasons, respectively, for a period of 37 days (rainy season = 15 days; dry season = 22 days). 72 sites (30.4%) were found to be positive (contain larvae of the *S. damnosum* complex) in total (a site positive in the two seasons is counted twice). Non-*Simulium damnosum* s.l. larvae were observed in some positive sites as well as in other sites that were negative for *S. damnosum* s.l. The number of *S. damnosum* s.l. breeding sites and the number of survey days, and the positive rates are shown in Table 1.

**Table 1:** Number of surveyed sites and survey days per season and *S. damnosum*-positive rates

Region	Rainy season		Dry season		Positive rate (%)	
	No. of surveyed sites (positive sites)	No. of days	No. of surveyed sites (positive sites)	No. of days	Rainy season	Dry season
Southwest	24 (12)	6	43 (32)	8	50	74.4
Northwest	15 (03)	3	35 (14)	5	20	40
North	42 (06)	6	78 (05)	9	14.3	6.4
Total	81 (21)	15	156 (51)	22	25.9	32.7

Breeding rates were higher in the dry season for the Southwest (74.4%) and the Northwest (40.0%), whereas they were higher in the rainy season for the North region (14.3%) (Table 1). A significant difference in breeding rates between the two seasons was observed for the Southwest region (*χ*^2^ = 4.07, *df* = 1, *P* = 0.04) but not for the Northwest (*χ*^2^ = 1.87, *df* = 1, *P* = 0.17) and North (*X*^2^ = 2.03, *df* = 1, *P* = 0.15) regions and not for the three regions combined (*χ*^2^ = 1.15, *df* = 1, *P* = 0.28).

**Fig. 2:**
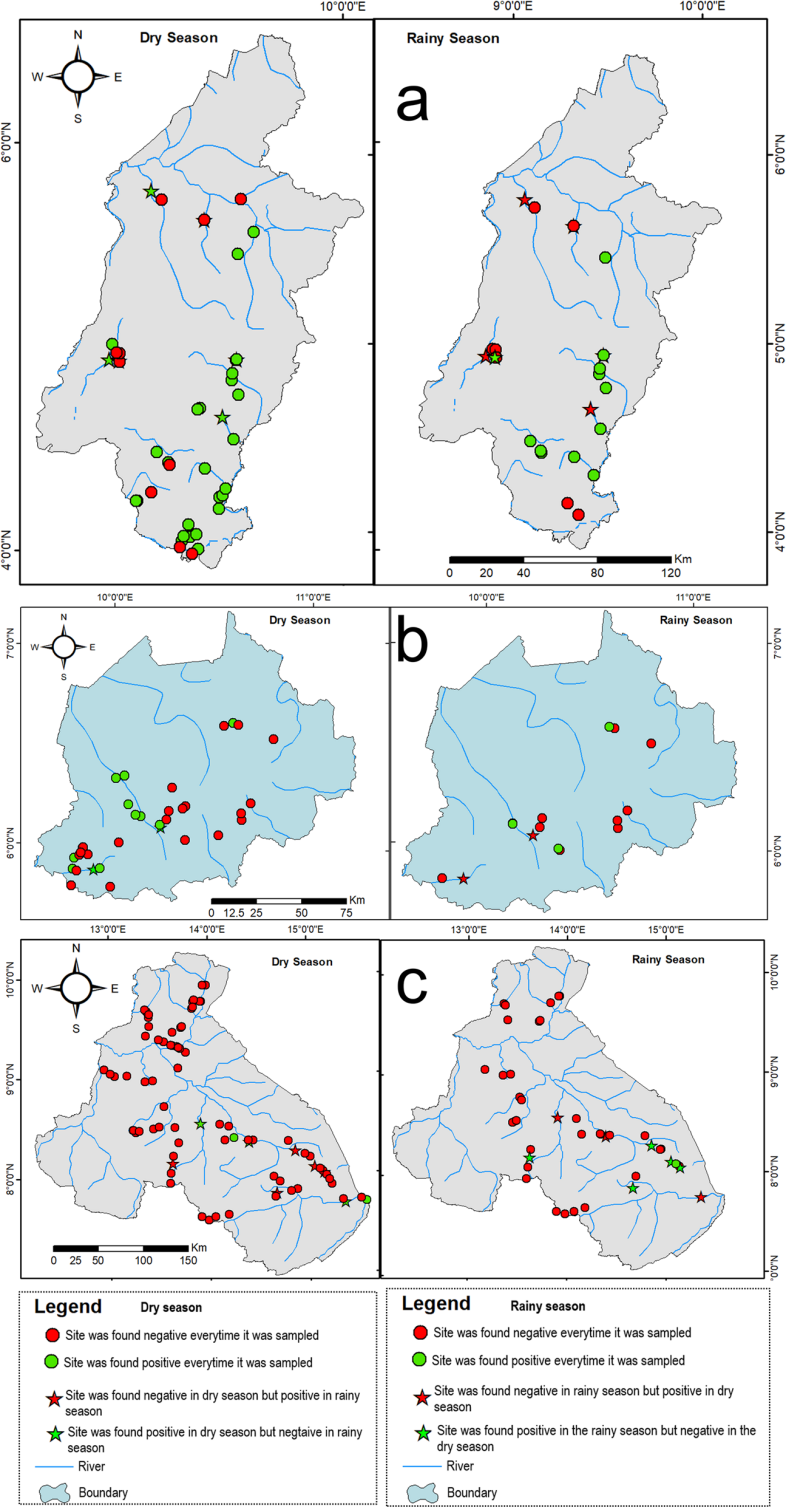
Breeding site maps showing sites that were sampled in the Southwest (**a**), Northwest (**b**) and North (**c**) regions

Out of the 68 sites that were visited during both sampling seasons (once per season), 16 (23.5%) were positive in one of the two seasons, while 14 (20.6%) and 38 (55.9%) sites were respectively positive and negative in both seasons. More of these sites sampled twice, and positive in one of two seasons, were positive in the dry season than in the rainy season for the SW (dry season [DS] = 6; rainy season [RS] = 1) and NW (DS = 02; RS = 00), while in the North region it was almost a tie (DS = 3; RS = 4), Fig. 2.

### Species distribution

**Fig. 3:**
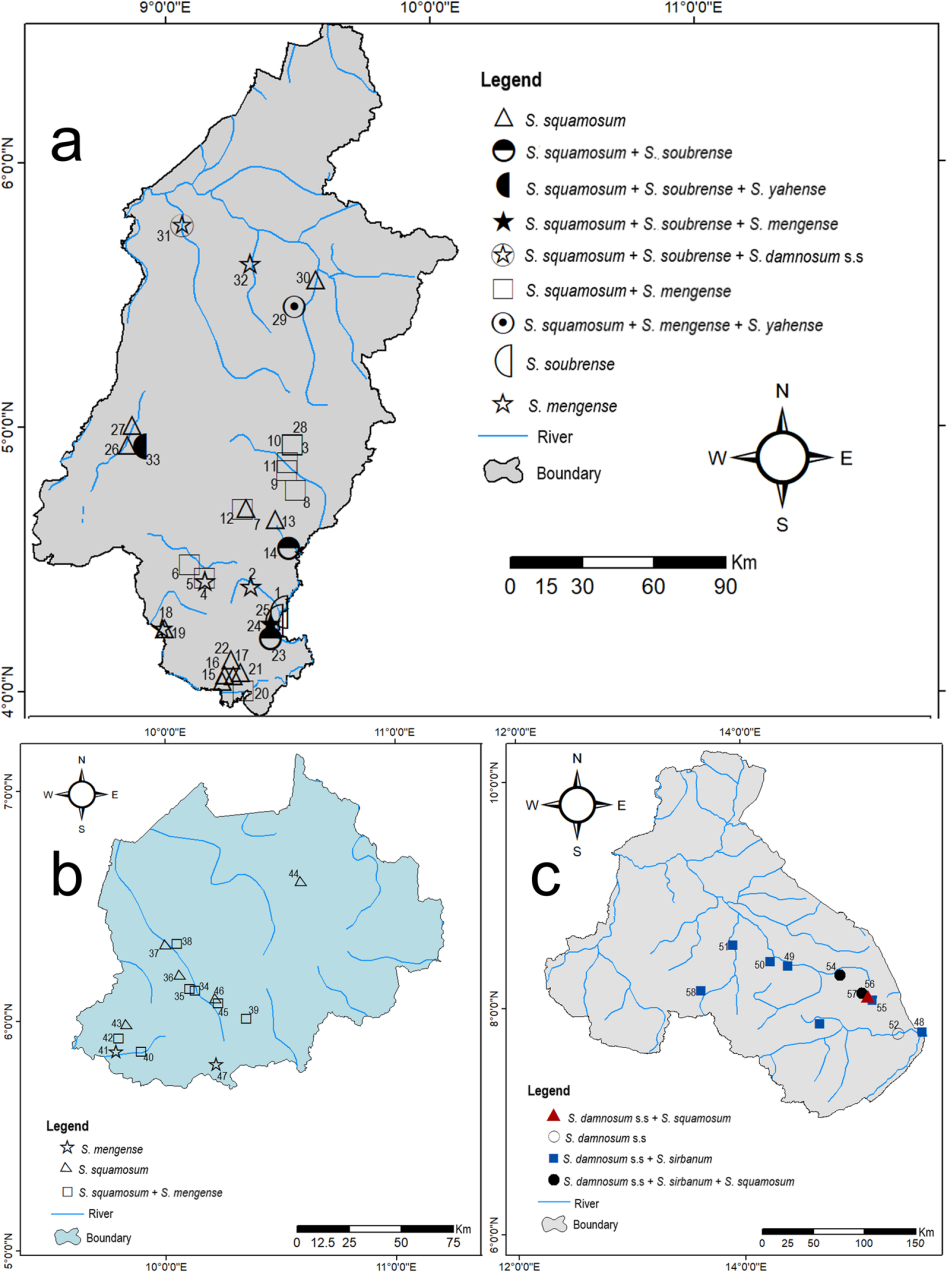
Species of the *S. damnosum* complex that were identified for both seasons in the Southwest (**a**), Northwest (**b**) and North (**c**) regions. *S. da/si* is plotted on the map as *S. sirbanum*. The numbers attached to the symbols correspond to the number (No.) in Table 3

Six different cytospecies were identified in this study. These include the forest species *S. squamosum*, *S. mengense*, *S. yahense* and *S. soubrense* Beffa form; and the savanna species *S. damnosum* s.s. and *S. sirbanum*. The Southwest and Northwest regions had predominantly forest species, while the North region had predominantly savanna species (Fig. 3, Tables 2 and 3).

**Table 2:** Prevalence of the vector species in the three regions between the two seasons

Species	Prevalence (%) in each region per season					
	Southwest		Northwest		North	
	DS (*n* = 449)	RS (*n* = 85)	DS (*n* = 158)	RS (*n* = 39)	DS (*n* = 68)	RS (*n* = 103)
*S. squamosum*	58.8	58.8	69.6	100.0	0.0	2.9
*S. mengense*	28.7	22.4	30.4	0.0	0.0	0.0
*S. soubrense* Beffa	12.0	16.5	0.0	0.0	0.0	0.0
*S. yahense*	0.2	2.4	0.0	0.0	0.0	0.0
*S. damnosum* s.s.	0.2	0.0	0.0	0.0	76.5	76.7
*S. sirbanum*	0.0	0.0	0.0	0.0	23.5	5.8
*S. damn/sirb*	0.0	0.0	0.0	0.0	0.0	14.6
Total	100.0	100.0	100.0	100.0	100.0	100.0

*DS:* dry season, *RS:* rainy season, *S. damn/sirb:* *Simulium damnosum*/*sirbanum*

**Table 3:** Breeding sites and species data for the three regions

No.	Sampling date	Nearest village	River	Geographical coordinates (UTM)			Species (No. identified)
				Easting	Northing	Altitude (m)	
*Southwest region*							
1	16-12-12	Yoke/Muyuka	Yoke	547345	475435	129	*S. so* Beffa (31)
	19-07-12	Yoke/Muyuka	Yoke	547345	475435	61	*S. so* Beffa (12)
2	16-12-12	Likoko	Njanje, trib. Yoke	535842	486125	103	*S. meng* (20)
	20-07-12	Likoko	Njanje, trib. Yoke	535836	486125	96	*S. meng* (08)
3	12-01-13	Wone	Nyale	553084	545542	259	*S. sq* (08), *S. meng* (06)
	23-07-12	Wone	Nyale	553060	545555	259	*S. sq* (01), *S. meng* (01)
4	16-12-12	Ebie	Sombe, trib. Meme	516640	488610	324	*S. meng* (07)
	20-07-12	Ebie	Sombe, trib. Meme	516638	488430	322	*S. meng* (01)
5	16-12-12	Newtown Bokosso	Sombe, trib. Meme	516085	489529	267	*S. sq* (01), *S. meng* (15)
	20-07-12	New Town Bokosso	Sombe, trib. Meme	516080	489530	287	*S. meng* (08)
6	16-12-12	Mofako Ekombe	Komborani, trib. Meme	509863	495106	49	*S. sq* (01), *S. meng* (02)
	20-07-12	Mofako Ekombe	Komborani, trib Meme	509889	495092	41	*S. sq* (01)
7	17-12-12	Big Massaka	Troteh	533238	519054	397	*S. sq* (01)
8	17-12-12	Big Massaka	Wubeh	532091	518311	302	*S. sq* (06), *S. meng* (15)
9	17-12-12	Diongo-Ikiliwindi	Menge	554244	526410	286	*S. sq* (06), *S. meng* (14)
	23-07-12	Diongo-Ikiliwindi	Menge	554276	526352	267	*S. sq* (11)
10	17-12-12	Konye	Mungo	553074	545480	259	*S. sq* (02), *S. meng* (01)
11	18-12-12	Bolo Mofako (Moboka)	Ilongo	550799	537835	224	*S. sq* (05), *S. meng* (16)
	23-07-12	Bolo Mofako (Moboka)	Ilongo	550764	537873	229	*S. sq* (02), *S. meng* (01)
12	18-12-12	Baduma	Nfem	550634	534408	242	*S. sq* (12), *S. meng* (08)
	23-07-12	Baduma	Nfem, trib. Mungo	550638	534402	242	*S. sq* (12)
13	18-12-12	Barombi/Kumba	Lake Barombi	545420	514141	310	*S. sq* (17)
14	18-12-12	Ediki	‘Kumba water’	551390	502168	45	*S. sq* (01)
	22-07-12	Ediki	‘Kumba water’	551346	502243	42	*S. so* Beffa (01), *S. sq* (11)
15	18-12-12	Ombe	Ombe river	531227	450539	161	*S. sq* (13)
16	18-12-12	Moliwe camp	Moliwe	527973	449231	178	*S. sq* (20)
17	18-12-12	Busumbu mile 2	Busumbu river	523555	446858	110	*S. sq* (21)
18	19-12-12	Sanje camp	Wongwe	499289	468751	25	*S. sq* (21)
19	19-12-12	Sanje Camp	Small Sanje	498586	468727	32	*S. meng* (12)
20	20-12-12	Mabeta Njanga, Besideschool	Mabeta	532374	442529	22	*S. sq* (29), *S. meng* (02)
21	20-12-12	Wotutu	Ewongo river	524383	449596	305	*S. sq* (12)
22	20-12-12	Tole camp	Tole/Ndongo	527089	455878	680	*S. sq* (05)
23	21-12-12	Pundu Balong	Mussaka	543506	464507	120	*S. sq* (11), *S. so* Beffa (01)
24	21-12-12	Meanja camp Muyuka	Meanja	543883	470714	36	*S. sq* (01), *S. so* Beffa (03), *S. meng* (03)
25	21-12-12	Pundu Balong	Mpobo	545410	471633	17	*S. so* Beffa (17)
26	11-01-13	Bulu camp, Mundemba	Mana	483832	545348	23	*S. sq* (02)
27	11-01-13	Korup National Park	Mana (upstream)	485735	553700	95	*S. sq* (20)
28	12-01-13	Konye	Mungo (downstream)	552972	545038	254	*S. sq* (14), *S. meng* (02)
29	12-01-13	Ayang	Mafeh/Manyu	553786	602859	175	*S. sq* (06), *S. ya* (01), *S. meng* (05)
	23-07-12	Ayang	Mafeh/Manyu	553979	602910	175	*S. sq* (01)
30	12-01-13	Bakebe	Mbo/Manyu	562573	614627	188	*S. sq* (14)
31	13-01-13	Akwen	Monaya	506692	637116	59	*S. da* (01), *S. sq* (15), *S. so* Beffa (02)
32	13-01-13	Talangaye	Badi (upstream)	535433	621119	100	*S. meng* (01)
33	21-07-12	Mundemba	Masopia	489311	544709	80	*S. so* Beffa (01), *S. sq* (11), *S. ya* (02)
*Northwest region*							
34	14-01-13	Bafut	Muchwine	624815	677903	809	*S. meng* (02)
	25-07-12	Bafut	Muchwine	624672	677938	834	*S. sq* (08)
35	14-01-13	Mantaah	Mezam	621810	678833	634	*S. sq* (15), *S. meng* (08)
36	14-01-13	Mbakong	Mbakong, trib. Mezam	617947	684623	595	*S. sq* (13)
37	14-01-13	Befang	Mawon	610995	699093	570	*S. sq* (19)
38	14-01-13	Ilium/wum	Mumeh	615724	700581	932	*S. sq* (11), *S. meng* (02)
39	15-01-13	Oshum	Teetip	601970	649257	1310	*S. sq* (11), *S. meng* (02)
	27-07-12	Oshum	Teetip	601970	649257	1310	*S. sq* (17)
40	15-01-13	Diche(ecolebilingue)/Widikum	Efigui, trib. Momo	585196	647860	515	*S. sq* (08), *S. meng* (06)
41	15-01-13	Begang/Widikum	Ndop, trib. Momo	586775	648788	535	*S. meng* (01)
42	15-01-13	Teze	Dudum bridge	587700	654906	604	*S. sq* (04), *S. meng* (10)
43	15-01-13	Andek	Fek	592388	660898	1112	*S. sq* (02)
44	16-01-13	Kamine	Kinte bridge2	676118	729715	1126	*S. sq* (13)
	26-07-12	Kamine	Kinte bridge 2	676163	729672	1126	*S. sq* (14)
45	17-01-13	Kedjom Keku	Fembvang	635822	671978	1088	*S. sq* (13), *S. meng* (03)
46	17-01-13	Kedjom Keku	Mih	635223	673246	1037	*S. sq* (01)
47	13-01-13	Bokwa	Bokwa	572408	572408	191	*S. meng* (14)
*North region*							
48	21-01-13	Bobdibo (Tchad border)	Mayo Vina	563129	857298	449	*S. da* (13), *S. si* (01)
49	22-01-13	Ntam	Mayo Djoulé	433764	923457	299	*S. da* (09), *S. si* (05)
50	24-01-13	Sarongari/Tchollire	Mayo Galke	416750	927945	208	*S. da* (07), *S. si* (09)
51	24-01-13	Benoue Forest Reserve	Benoue	380102	944677	256	*S. da* (10), *S. si* (01)
52	21-01-13	Touboro	Mayo Vina bridge	539810	856294	479	*S. da* (13)
53	06-09-12	Kaou-Bidam	Ri	464129	866682	601	*S. da* (20), *S. si* (02), *S. da*/*si* (01)
54	06-09-12	Kodjong	Mbe	484516	913499	401	*S. da* (12), *S. si* (01), *S. da*/*si* (01), *S. sq* (01)
55	07-09-12	Mbakana I	Ndah	516313	889365	613	*S. da* (24), *S. da*/*si* (07)
56	07-09-12	Garang Pont	Wah	511770	893093	501	*S. da* (06), *S. sq* (01)
57	07-09-12	Laoudougoy	Mayo Laoudougoy	506113	895565	557	*S. da* (08), *S. si* (02), *S. da*/*si* (02), *S. sq* (01)
58	08-09-12	Mbam	Mayo Mbam	349115	900620	398	*S. da* (09), *S. si* (01), *S. da*/*si* (04)

The coordinates for the rainy season samples were converted from decimal degree to UTM (values rounded to the nearest whole number) using the free online resource https://www.latlong.net/lat-long-utm.html using the following specifications: UTM Zone for the Southwest and Northwest regions = 32 N; UTM Zone for the North region = 33 N. Though the species status of *S. da/si* is not yet known, it is assumed to be *S. sirbanum* on the maps, for convenience’s sake.

*S. sq:* *Simulium squamosum*, *S. meng:* *Simulium mengense*, *S. ya:* *Simulium yahense*, *S. so* Beffa: *Simulium soubrense* Beffa form, *S. da:* *Simulium damnosum* s.s., *S. si:* *Simulium sirbanum*, *S. da/si:* *Simulium damnosum*/*sirbanum*, *trib.:* tributary of

Five of the six species identified in this study occurred in the Southwest region, making it the region with the highest species diversity (*S. squamosum*, *S. mengense*, *S. soubrense*, *S. yahense* and *S. damnosum* s.s.), followed by the North region with three species (*S. damnosum* s.s., *S. sirbanum*, *S. squamosum*) and then the Northwest region with two (*S. squamosum* and *S. mengense*) (Table 2 and Fig. 3). *Simulium soubrense* Beffa form was mostly observed in the Yoke/Muyuka and surrounding breeding sites in both seasons.

No marked changes in species distribution were observed in the breeding sites that were sampled in the two seasons. Some species were noted to be absent in one season but present in the other season at the same sites. We did not, however, observe any association between a particular season and the presence/absence of a species, except for the presence of the individual larvae that could not be placed simply as *S. sirbanum* or *S. damnosum* s.s. that were only observed in rainy season samples of the North region (Tables 2 and 3) at a frequency of 0.146 (*n* = 103).

In the Southwest and Northwest regions, *S. squamosum* was the most prevalent species at nearly constant proportions in both seasons. *Simulium mengense* was more common during the dry season, but was totally absent in all identified rainy season samples from the Northwest region. Similarly, in the North region, *S. damnosum* s.s. was the most prevalent species in the two seasons, while the prevalence of *S. sirbanum* dropped drastically in the rainy season accompanied by observation of the *Simulium damnosum*/*sirbanum* cytotype (Table 2).

## Discussion

### Breeding sites of *S. damnosum* s.l.

Sampling of the aquatic stages of members of the *S. damnosum* complex is important in mapping the breeding sites of the vectors. This is necessary for determining the distribution of the vectors, selection of vector collection sites and sentinel communities for entomological and epidemiological evaluations, respectively. Furthermore, it helps in identifying areas of possible onchocerciasis transmission and understanding the local epidemiology of the disease. Hence, the breeding site survey is a key step in the monitoring and evaluation of the different phases of onchocerciasis elimination [10]. Except for the study by Traore-Lamizana et al. [15], the present study has mapped more *S. damnosum* s.l. breeding sites and species distribution in Cameroon than any previous study. It provides an updated and more comprehensive survey encompassing several regions and vegetation types. This survey did not show any challenges peculiar to any vegetation type, contrary to reports of difficulty in searching for breeding sites in forest areas [12].

Breeding sites of *S. damnosum* s.l. were more prevalent in the Southwest region than the Northwest and North regions, and more in the Northwest than the North region. This is due to the perennial nature of the rivers in the Southwest region and the dense tropical rainforest in this region, which provides continuous nutrient-rich water for the development of *Simulium* larvae. Whereas most streams/rivers in the North region are seasonal.

Similar to earlier observations [15, 18] most rivers in the North region, except the few perennial rivers, were reduced to trickles or pools or completely dried out during the dry season. Breeding in the North region occurs such that during the rainy season, it is almost exclusively in the smaller seasonal streams (tributaries) while the perennial and semi-perennial (larger streams/rivers) streams/rivers are overflooded. Breeding gradually begins in the perennial and semi-perennial streams/rivers when the water descends to intermediate levels, during which time the seasonal streams have begun drying out. By the middle of the dry season, breeding occurs exclusively in the perennial rivers as all other streams/rivers would have dried off.

Hence, the number of breeding sites (and therefore, the risk of onchocerciasis transmission) in the North region may be more important during, either the rainy or the dry season, depending on whether the river is seasonal, semi-perennial (larger streams/rivers) or perennial. Variations in seasonal breeding were observed in all three regions, thereby highlighting the importance of proper timing of breeding site surveys and other entomological studies.

Generally, it is considered that the highest transmission period occurs during the rainy season, when water flows in all the riverbeds. However, this is not always the case as we observed in the Southwest and Northwest regions. Our data showed that in these regions, the proportion of breeding sites increased during the dry season, compared with the rainy season. In fact, comparing the potential breeding sites that were visited in both seasons in these two regions, it was observed that all the rainy season positive breeding sites were again positive during the dry season, while several sites that were negative during the rainy season became positive during the dry season. Achukwi et al. [24] also observed higher biting during the dry season in the Vina du Sud in Cameroon, indicating increased breeding during the dry season.

Higher breeding in the dry season compared with the rainy season could be explained by the flooding of some of these rivers during the rainy season that leads to the sweeping away of potential larval supports (e.g. vegetation, trapped fallen leaves, fallen tree branches, etc.) by the high water current, and/or the submergence of breeding sites by the overflooding turbid dirty waters [31].

On the other hand, during the dry season (or early dry season for the large but seasonal rivers in the North region), the water levels drop, exposing submerged rocky beds and creating rapids, and the waters are less turbid, therefore favouring breeding. Hence, seasonal changes in water level [1, 31], turbidity [31] and availability of rapids and submerged vegetation are important factors in the breeding and distribution of black fly vectors.

### Distribution of *S. damnosum* s.l.

The distribution of *S. damnosum* s.l. species was according to the bio-climate of the regions, with the North (Sudan savanna) region harboring the savanna species (*S. damnosum* s.s. and *S. sirbanum*) and the Southwest (rainforest—*S. squamosum*, *S. mengense*, *S. soubrense* Beffa form, *S. yahense*, *S. damnosum* s.s.) and Northwest (mixed forest–savanna—*S. squamosum* and *S. mengense*) regions harbouring the forest species. The high diversity of species found in the Southwest region could be due to the abundant breeding sites that provide a wide range of favourable microhabitat conditions for the adaptation of the diverse black fly species of different ecological preferences. This area has been suggested to be one of the centres of greatest diversity of the *S. damnosum* complex in Africa, where Mount Fako and the associated forests may play an important role in their evolution [32].

Based on its predominance in the Southwest and Northwest regions, *S. squamosum* could be playing a major role in onchocerciasis transmission in these regions, assisted by *S. mengense*. Similarly, the main vector in the North region is *S. damnosum* s.s., followed by a small *S. sirbanum* proportion which increases during the dry season [16] and may play a minor role in transmission in this area. The species and vectorial status of the *S*. *damnosum*/*sirbanum* cytoform is yet to be elucidated.

Various species of the *S. damnosum* complex present different transmission efficiencies [5] hence, increased diversity may lead to complex epidemiology of onchocerciasis. For example, *S. squamosum* and *S. mengense* (the major onchocerciasis vectors in the Southwest and Northwest regions) have been shown to present bilateral blindness similar to what has previously been established for savanna vectors [33]. Generally, there were no seasonal changes in species composition in the different regions. However, at some breeding sites, different species were observed in different seasons. This could be due to seasonal dispersal of species or seasonal variation in physiochemical composition of the rivers that do not favour survival of certain species.

This study has shown how widespread *S. mengense* is, since its first description from the Menge River in the Southwest region [34]. Furthermore, contrary to previous studies [17, 19, 35] which only recorded *S. squamosum* in Yoke/Muyuka and surrounding breeding sites, the present study observed a predominance of *S. soubrense* Beffa form in the area. This is the first report of the *S. soubrense* Beffa form in Cameroon. Longitudinal studies would be required to determine whether these changes in cytospecies composition in the Yoke/Muyuka breeding site and its environs are permanent or temporary.

The observation of *S. soubrense* in the Southwest region is probably due to the recent spread of this cytospecies from neighbouring Nigeria where it has previously been reported [21], especially as all previous studies in this area did not record this species [17, 19, 35]. This finding has implications for the epidemiology of onchocerciasis in this region given that *S. soubrense* Beffa form seems a closer relative to the savanna than the forest cytospecies [36], and it is a more efficient vector than most forest and savanna vectors, including *S*. *squamosum* [5], which has been the main cytospecies in this area [17, 19, 35].

## Conclusion

Although not exhaustive, this study provides comprehensive, updated and representative data on breeding sites and species distribution in the Southwest, Northwest and North regions of Cameroon. The difference in positive breeding sites with respect to seasons emphasizes the importance of carrying out breeding site surveys in both rainy and dry seasons. This study has also demonstrated the feasibility of breeding site surveys, in both rainy and dry seasons, in a rainforest zone. Also, this study has shown that not all potential breeding sites are actual breeding sites. The report of *S. soubrense* in the Southwest region indicates changes in species composition over time and could affect the epidemiology of onchocerciasis in this area.

## Supplementary Information

**Additional file 1: Fig. S1.** Relief map of the North region.

## Data Availability

The datasets analysed during the current study are available from the corresponding author on reasonable request.

## Abbreviations

DS: Dry season
RS: Rainy season
REMO: Rapid epidemiological mapping of onchocerciasis
SW: Southwest region
NW: Northwest region
N: North region
