# Supplementary figures and images for: A cross-sectional study of Simulium damnosum sensu lato breeding sites and species distribution in Sudan savanna, mixed savanna–forest and rainforest regions in Cameroon
Source: Parasit Vectors. 2022 Oct 21;15:382. doi: 10.1186/s13071-022-05462-w (PMC9587638; doi:10.1186/s13071-022-05462-w)

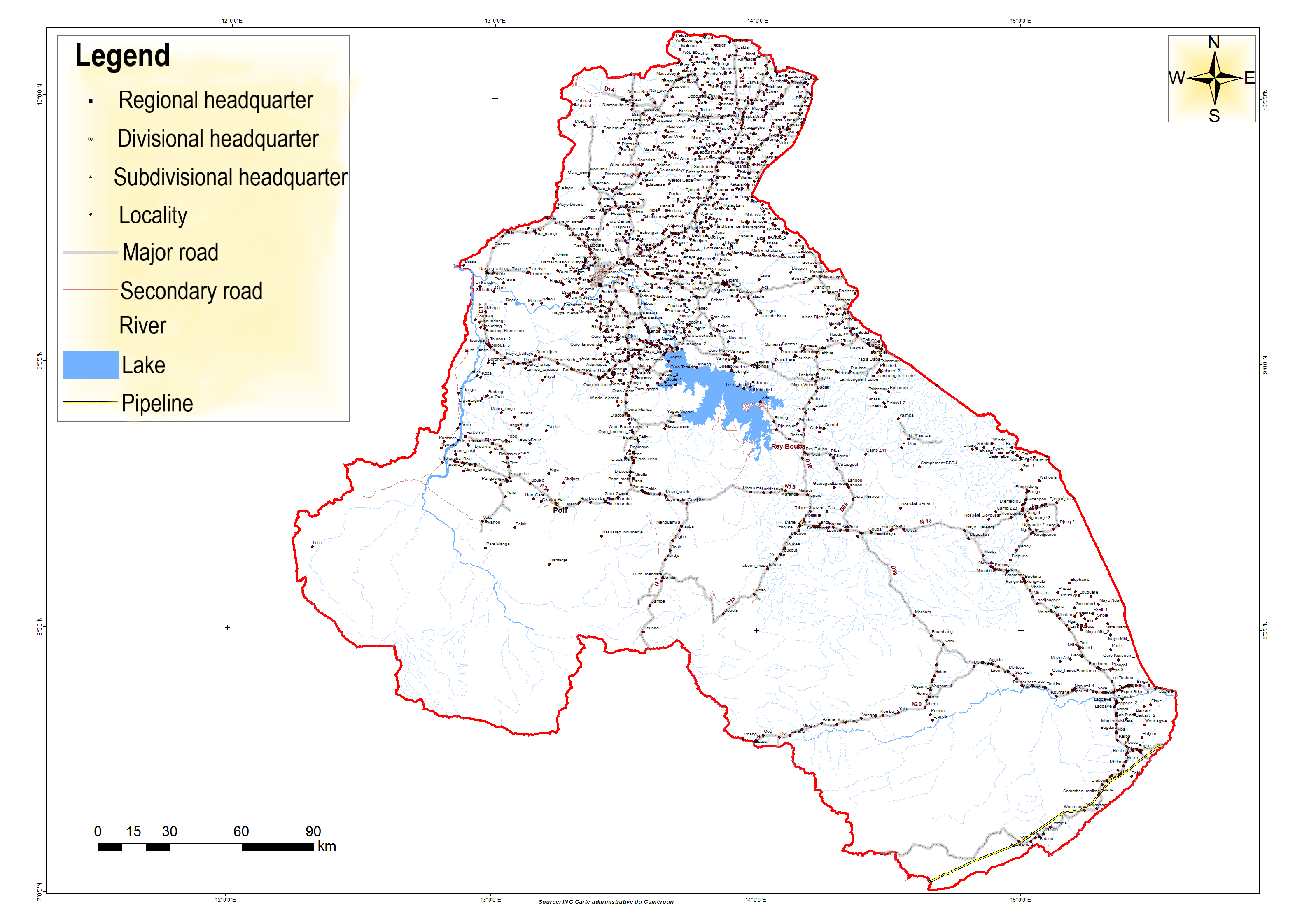

Supplement: Supplementary file 1 — Additional file 1: Fig. S1. Relief map of the North region. [file 13071_2022_5462_MOESM1_ESM.tiff]
